# Managing Hashimoto’s Encephalopathy: A Case Report

**DOI:** 10.7759/cureus.80467

**Published:** 2025-03-12

**Authors:** Gertrūda Dagytė, Sofija Mančinskaja, Jurgita Krasauskienė

**Affiliations:** 1 Internal Medicine, Lithuanian University of Health Sciences, Kaunas, LTU; 2 Internal Medicine, Palliative Care and Family Health Center, Klaipėda, LTU; 3 Neurology, Lithuanian University of Health Sciences, Kaunas, LTU; 4 Neurology, Klaipėda’s University Hospital, Klaipėda, LTU

**Keywords:** encephalopathy, hashimoto encephalopathy, hashimoto’s thyroiditis, steroid-responsive encephalopathy associated with autoimmune thyroiditis, unexplained encephalopathy

## Abstract

Hashimoto’s encephalopathy (HE), also known as steroid-responsive encephalopathy associated with autoimmune thyroiditis (SREAT), is a rare condition associated with thyroid autoimmunity. Characterized by cognitive decline, seizures, and neuropsychiatric symptoms, HE remains poorly understood and poses diagnostic challenges due to its variable criteria presentations and overlap with other conditions. Diagnosis typically involves clinical criteria, emphasizing encephalopathy in conjunction with positive thyroid antibodies, exclusion of alternative causes, and responsiveness to corticosteroid treatment.

We present the case of a 76-year-old male patient admitted with acute gait disturbance and disorientation, later developing seizures and respiratory failure. Initial imaging demonstrated diffuse cortical atrophy, nonspecific white matter ischemic changes, and a right frontal meningioma. While infections and metabolic causes were ruled out, significantly elevated anti-thyroid peroxidase (ATPO) antibodies suggested HE. Corticosteroid therapy was administered with methylprednisolone pulse treatment (1 g/day for three days), leading to substantial neurological improvement and stabilization. Despite some residual ataxic symptoms, the patient's overall functional and cognitive status showed significant recovery.

This case emphasizes the importance of considering HE in patients with unexplained encephalopathy and positive thyroid antibodies. Early intervention with corticosteroids can be vital in preventing lasting neurological damage. Continued research into HE is essential to refine diagnostic criteria, enhance differential diagnosis, and establish evidence-based treatment protocols that optimize patient outcomes.

## Introduction

In 1966 Dr. Lord Brain and his colleagues were the first to describe a patient with Hashimoto's encephalopathy (HE). They reported a man who presented with various neurological symptoms: cognitive dysfunction, hemiplegia, hemianopia, sensory disturbance, hallucinations, and stroke-like episodes. The patient’s cerebrospinal fluid test showed high levels of protein, and these results and symptoms were linked to the patient’s prior diagnosis of Hashimoto’s thyroiditis (HT) [1]. Currently, HE remains poorly understood, and its pathogenesis continues to be uncertain. Available evidence points to autoimmune etiology; however, it is yet to be proven [2].

The diagnosis of this rare disorder is guided by diagnostic criteria developed by Dr. Pablo E. Castillo and colleagues [3]. These criteria were shaped by an analysis of prior clinical studies and publications. To meet the diagnostic threshold, patients must exhibit encephalopathy characterized by cognitive impairment and at least one neuropsychiatric feature, such as hallucinations, delusions, myoclonus, seizures, or focal neurological deficits. The presence of serum thyroid antibodies is required, with patients maintaining either a euthyroid state (thyroid-stimulating hormone (TSH) 0.3-5.0 mIU/L) or mild hypothyroidism (TSH 5.1-20.0 mIU/L), as thyroid dysfunction alone does not account for the encephalopathy. Exclusion criteria eliminate other possible causes, such as infections, toxic or metabolic conditions, or neoplastic processes, as confirmed through blood, urine, and cerebrospinal fluid (CSF) analyses. Furthermore, no paraneoplastic autoantibodies, including voltage-gated calcium or potassium channel antibodies, should be present, and neuroimaging should reveal no vascular, neoplastic, or structural abnormalities that could explain the symptoms. Importantly, patients must show complete or near-complete neurological recovery following corticosteroid therapy, highlighting the treatment-responsive nature of this condition [3]. For this reason, some call HE a steroid-responsive encephalopathy associated with autoimmune thyroiditis (SREAT) [4]. In the study of 251 SREAT cases, 91% of treated patients improved, with steadily declining levels of antithyroid antibodies following immunosuppressive therapy [5]. However, some reports argue about the efficacy of treatment [3].

In 2016, Dr. Giacomo Montagna and his colleagues developed a comprehensive treatment protocol for HE, emphasizing corticosteroid therapy as the primary intervention due to its effectiveness in reducing inflammation and managing symptoms. For initial treatment, they recommend high-dose intravenous methylprednisolone at 500-1000 mg daily for three to five days or oral prednisone at 1-2 mg/kg per day, gradually tapered to minimize side effects and reduce relapse risk. In cases where corticosteroids alone are insufficient, additional immunomodulatory agents such as azathioprine, methotrexate, cyclophosphamide, mycophenolate, hydroxychloroquine sulfate, or rituximab may be introduced to enhance the therapeutic response. For patients unresponsive to these treatments, alternative options, including intravenous immunoglobulin (IVIG) or plasmapheresis, can be considered, particularly for severe or refractory symptoms. This multi-tiered approach allows for personalized and effective HE management, aiming to improve patient outcomes through tailored therapy [6].

Hashimoto's encephalopathy remains a controversial disorder, with many believing there is a link between HT and HE. However, some argue that there is no evidence of a pathogenic role of the antibodies. It remains to be proven whether central nervous system tissue damage is directly caused by thyroid autoantibodies. For now, these antibodies can only be used as disease markers. Additionally, some state that HT is not the cause of the illness because elevated thyroid antibodies can be seen in many other diseases that do not result in neurological symptoms [2, 3].

Even an affected population of HE is not precise and relies on speculations. The disorder is estimated to affect 2.1 per 100,000 individuals in the general population [7, 8]. Hashimoto's encephalopathy usually affects women more than men (ratio of 4:1) and is commonly seen in adults rather than pediatric patients. The prognosis varies due to some patients experiencing relapses or requiring longer treatment [9].

## Case presentation

A 76-year-old Caucasian male patient presented to the emergency care with acute impaired gait and disorientation, marked by an inability to handle a phone despite having driven a vehicle that morning. Patients’ families reported a history of two to three episodes every six months of transient disorientation, inappropriate behavior, and incoordination with symptoms resolving within several days, alongside a gradual cognitive decline over the past six months. 

On initial examination, the patient was conscious, disoriented, and maintained eye contact, though exhibited motor aphasia and had longstanding strabismus in the left eye. The neurological assessment revealed symmetrical limb movement without motor deficit, normal deep tendon reflexes, and no pathological reflexes. Significant ataxia in an upright position was observed, rendering the patient unable to walk. A full-body orthostatic tremor was observed. Vital signs were stable with high normal blood pressure at 138/86 mmHg. A brain CT revealed enlarged lateral ventricles, diffuse cortical atrophy, and signs of nonspecific white matter ischemic changes. Laboratory investigations were unremarkable, leading to the patient’s admission to the neurology department for further evaluation.

**Figure 1:**
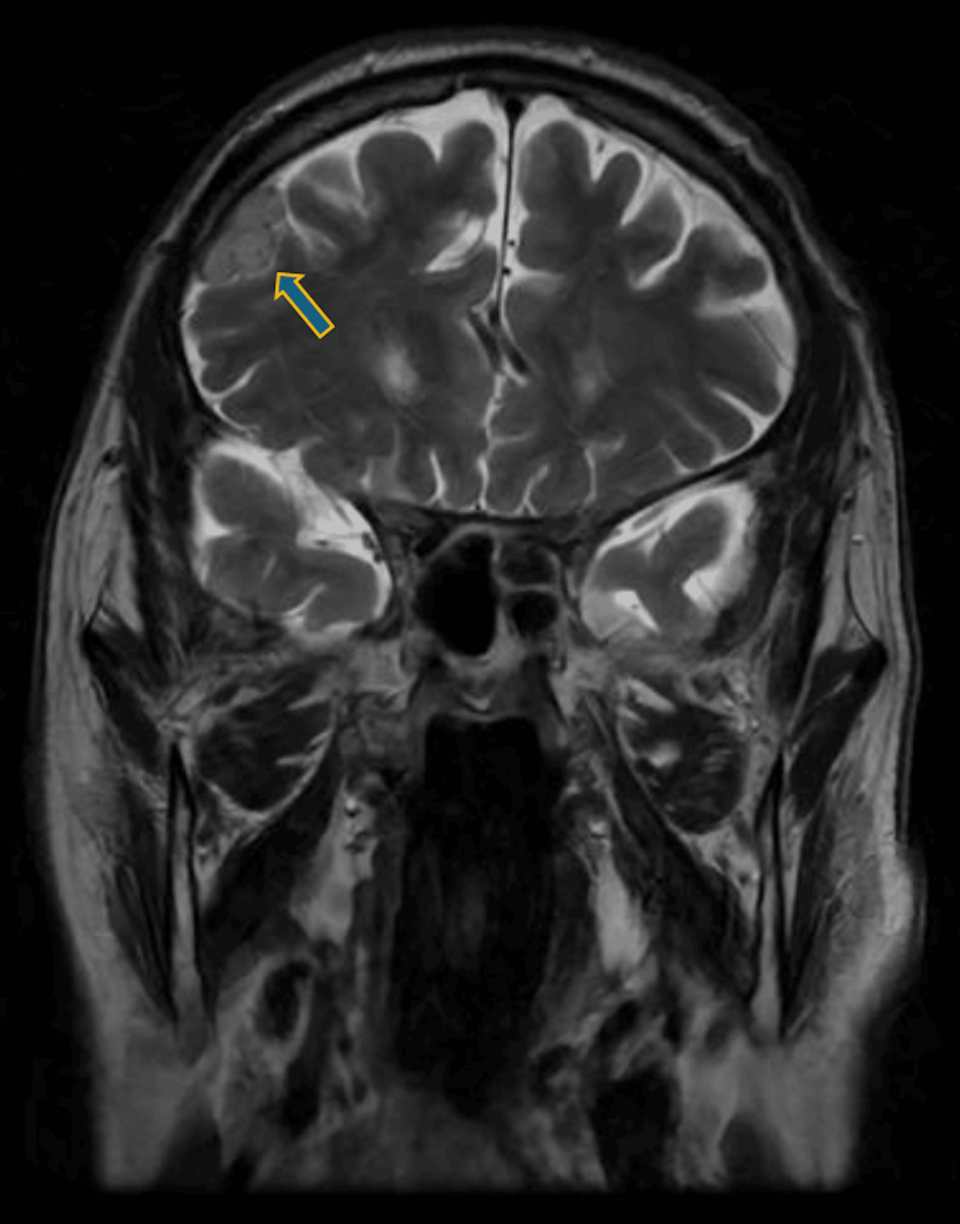
Brain MRI (T2 coronal) without contrast showed a right frontal lobe meningioma.

**Figure 2:**
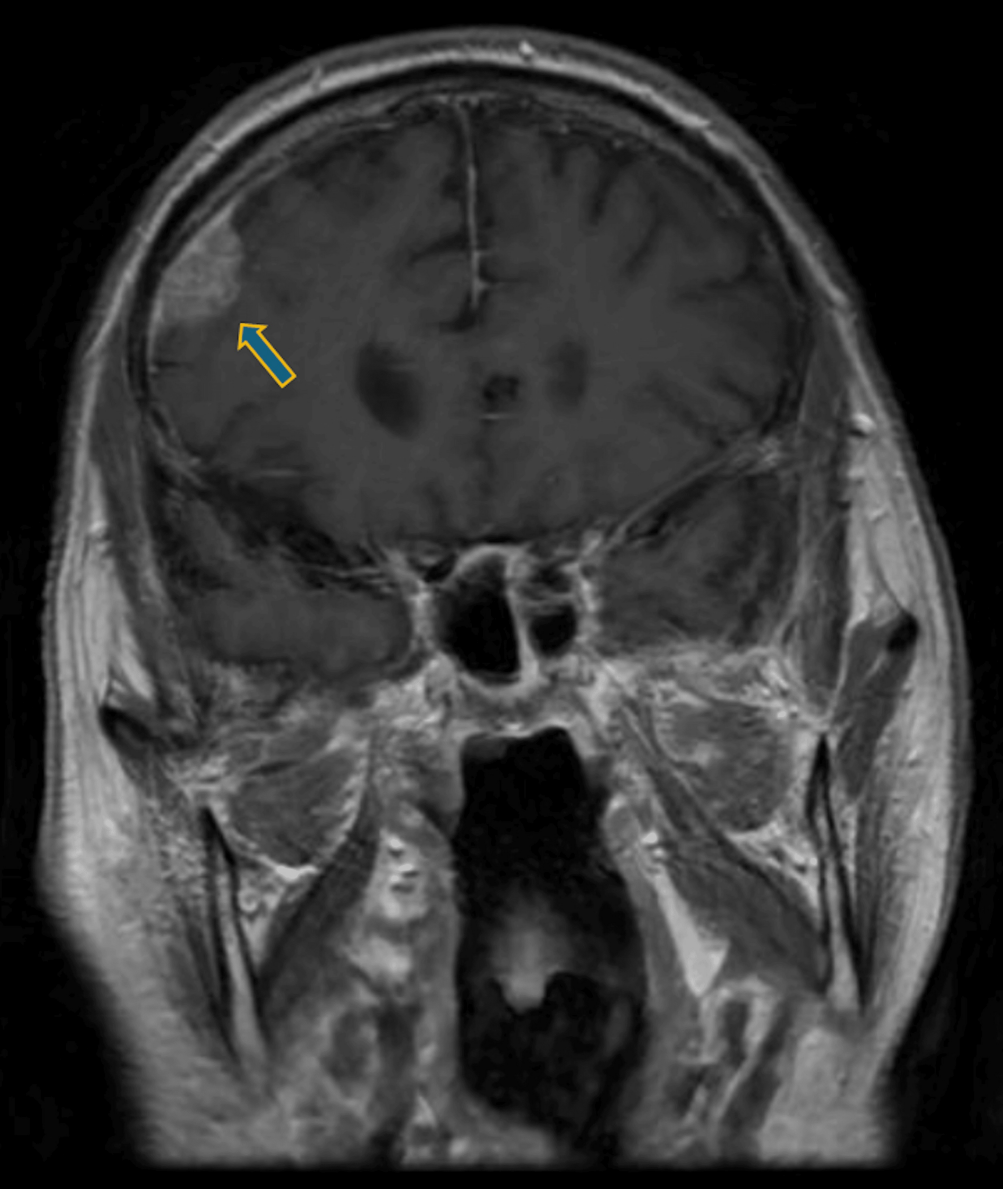
Brain MRI (T1 coronal) with contrast showed a right frontal lobe meningioma.

**Figure 3:**
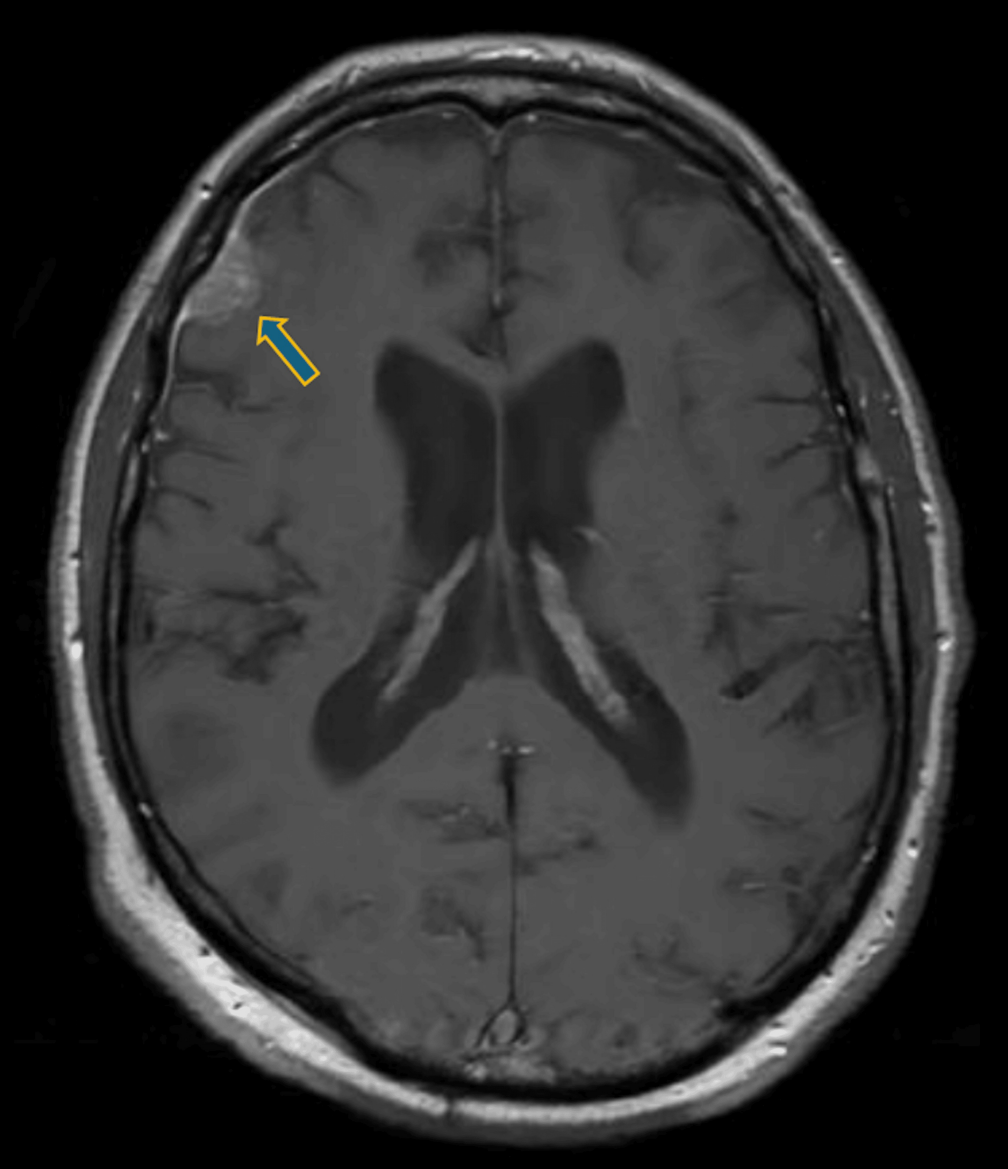
Brain MRI (T1 axillar) with contrast showed a right frontal lobe meningioma and enlarged ventricles.

**Figure 4:**
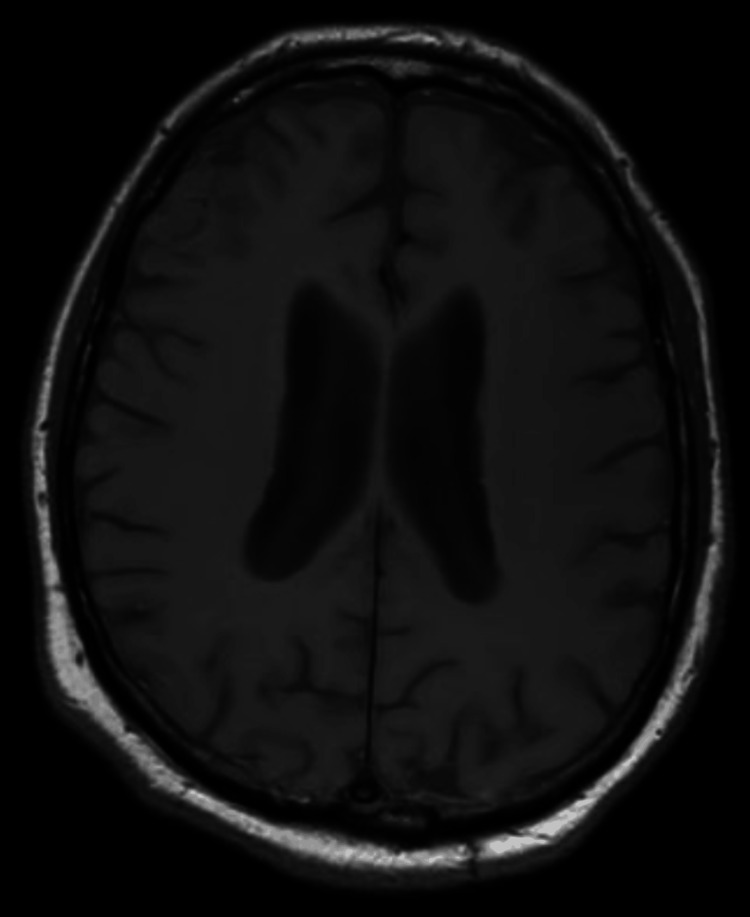
Brain MRI (T1 axillar) without contrast showed enlarged lateral ventricles.

In the neurology department, the patient remained conscious but disoriented with persistent motor and sensory aphasia and stuttering. He exhibited agitation and anxiety. The patient fell backward when standing or seated, adopting a wide stance, and demonstrated difficulty understanding commands during coordination tests, despite symmetrical and adequate limb muscle strength. Repeated blood tests remained normal. That night the patient experienced visual hallucinations, reporting erroneous perception of movement within the stationary cupboard, and the following day became increasingly disoriented. After a psychiatrist's consultation, the patient was diagnosed with unspecified delirium and recommended neuroleptics (tiapride, haloperidol). A cranial MRI (Figures 1-4) revealed grade II internal hydrocephalus, vascular foci in the pons and both hemispheres and atypical meningioma in the right frontal lobe, necessitating differentiation from lymphoma and mantle metastasis.

A multidisciplinary consultation was sought, involving a speech therapist, hematologist, and endocrinologist. The speech therapist identified dysarthria and motor aphasia, accompanied by acoustic-mnemic aphasia. The hematologist recommended a biopsy and full-body CT to investigate potential lymphoma or a primary oncological process. The full body CT scan showed a retrosternal goiter and parenchymal kidney cysts (Bosniak I) with no evidence of malignancy; however, the patient refused a brain biopsy. The endocrinologist diagnosed non-toxic multinodular goiter and ordered anti-thyroid peroxidase (ATPO) and anti-thyroglobulin (anti-TG) tests to screen for thyroid carcinoma.

The CSF analysis showed elevated protein concentration (1.1 g/L), a negative test for tick-borne encephalitis, and no evidence of lymphoproliferative disorders on flow cytometry. An EEG was also unremarkable. Given the unclear etiology, 14-3-3 protein was tested in the CSF to rule out Creutzfeldt-Jakob disease (CJD), with results expected in three to four weeks.

On the sixth day of hospitalization, the patient experienced three generalized tonic-clonic seizures, controlled with intravenous diazepam (10 mg); however, his Glasgow Coma Scale (GCS) dropped to nine (E2, V2, M5). He developed a low-grade fever, oxygen saturation decreased to 85% despite 10 L/min oxygen supply, and sepsis was indicated by leukocytosis (16.11 x 109/L) and elevated C-reactive protein (291.7 mg/L). A chest X-ray revealed focal inflammatory infiltration in the right middle and lower lung lobes, prompting meropenem administration. A repeated brain MRI showed no significant changes. 

Given the clinical course, anti-N-methyl-d-aspartate (NMDA) autoimmune encephalitis secondary to thyroid carcinoma or HE (SREAT) was suspected. Despite an initial improvement with decreased C-reactive protein (115 mg/L) and fever lysis, the patient was still somnolent.

Consultation with the endocrinologist and internal medicine specialist led to the diagnosis of HE, exacerbated by aspiration pneumonia. Methylprednisolone pulse therapy was recommended; however, in the presence of severe infection and the absence of vital indications, the steroid treatment was postponed until the infection resolved. The next day, despite regular administration of valproate 500 mg twice per day, the patient experienced another seizure, with GCS dropping to five (E2, V2, M1), and he progressed to type I hypoxemic respiratory failure (respiratory rate (RR) 40 breaths/minute, oxygen saturation 95% with 15 L/min supplemental oxygen, and an arterial blood test which reported partial pressure of carbon dioxide (PaCO_2_) 54.5 mmHg; partial pressure of oxygen (PaO_2_) 52.1 mmHg; pH: 7.360; bicarbonate (HCO_3_): 27.2 mmol/L), necessitating transfer to the ICU.

The antibacterial regimen was adjusted following the identification of hospital-acquired pneumonia with culturally proven *Acinetobacter baumannii *in the respiratory tract, leading to the replacement of meropenem with colistin. A subsequent autoimmune encephalitis antibody panel returned negative. Although elevated ATPO levels (>1000 IU) alone are not a definitive diagnostic criterion for HE, methylprednisolone pulse therapy (1 g/day for three days) was initiated based on suspicion. This treatment resulted in gradual clinical improvement, allowing the patient to be transferred back to the neurology department, and the diagnosis of HE was finally confirmed.

On day 17, the chest X-ray showed improvement. By day 25, the CJD test came back negative, and following intravenous methylprednisolone pulse 1 g per day therapy, the patient demonstrated significant clinical improvement, ambulatory within the ward, though with residual vestibulotoxic syndrome and encephalopathic symptoms. The final diagnosis concluded HE, autoimmune thyroiditis, hypothyroidism, acute symptomatic seizure, pulmonary obstruction, and sepsis.

Cranial MRI findings suggested a meningioma in the right frontal lobe, warranting follow-up imaging and neurosurgical consultation in one to two months. Methylprednisolone was transitioned to prednisolone 60 mg/day, with a planned dose reduction of 5-10 mg per week after three to four weeks, followed by gradual withdrawal over three months. Additionally, valproate 500 mg twice daily was prescribed, with a recommendation to monitor serum valproic acid concentration after one month. Levothyroxine was not indicated due to low TSH; however, selenium supplements (200 mg/day) were recommended. A follow-up appointment with an endocrinologist is planned in one to two months.

After 32 days, the patient was discharged to the physical rehabilitation department for further recovery. Upon discharge, the patient presented with vestibulotoxic syndrome, resulting in gait instability, impaired postural control, and reduced functional independence in activities of daily living. However, no dysphagia nor focal neurological deficits were present, and he had a normal respiratory status.

## Discussion

While this case report offers valuable insight into the presentation and management of HE, several limitations must be considered. Firstly, the diagnosis of HE remains largely clinical, based on criteria such as those proposed by Dr. Pablo Castillo et al. [3], which aid in guiding diagnosis but may still lead to uncertainty, especially when the condition coexists with other neurological or systemic diseases, as seen in this patient. The presence of multiple comorbidities complicates the clinical picture and raises the possibility of overlapping etiologies contributing to the patient’s symptoms.

In cases of HE, neuroimaging findings can be particularly useful in supporting the diagnosis. The most common MRI finding is diffuse cortical-subcortical white matter hyperintensity, observed in approximately 50% of cases with positive MRI results. T2-weighted/fluid-attenuated inversion recovery (FLAIR) hyperintensity in the temporal lobes is also common, appearing in about 20% of cases, followed by basal ganglia and/or thalamic hyperintensity in around 10% of patients. Brainstem involvement is seen in approximately 6% of cases, while spinal cord involvement, though rare, has been documented as T2 hyperintensity in the cervical and thoracic spinal segments, specifically at T5-T6. Together, these MRI findings help to establish a neuroimaging profile for SREAT, aiding in its differentiation from other conditions; however, seronegative autoimmune encephalitis, which is also responsive to steroids, cannot be definitively ruled out [10].

Additionally, the reliance on elevated anti-thyroid antibody levels as a key diagnostic marker is problematic, as these antibodies can be elevated in other conditions, such as HT, which limits their specificity for HE. These limitations underscore the difficulty of diagnosing HE in the presence of confounding factors and highlight the need for more definitive diagnostic tools.

Finally, even though HT is the most common autoimmune thyroid disease, HE remains an unusual and vaguely understood disorder. Despite its association with HT, HE can occur without thyroid dysfunction, adding to the diagnostic challenge. Early diagnosis and prompt treatment are crucial to prevent permanent neurological damage. However, much remains unknown, and further research is needed to understand HE better and develop standardized treatment protocols.

## Conclusions

This case suggests how important it is to raise awareness of HE among clinicians, so if unexplained encephalopathy is diagnosed, especially in the presence of longstanding thyroid health issues, HE would be in the differential diagnosis. Early recognition and immediate treatment are crucial for significant improvement and prevention of irreversible neurological damage. Additionally, primary treatment should be considered with glucocorticoids; if not tolerated, physicians should include alternative immunosuppressive therapies such as IVIG or plasmapheresis, though complete recovery is not guaranteed. Further research is essential to better understand the pathogenesis of HE and to establish more standardized treatment protocols.
